# Pharmacological Investigation of a Novel Resveratrol-like SIRT1 Activator Endowed with a Cardioprotective Profile

**DOI:** 10.3390/molecules30224378

**Published:** 2025-11-13

**Authors:** Leonardo Carbonetti, Simone Brogi, Rosarita D’Orsi, Marco Lessi, Vincenzo Calderone, Lara Testai, Fabio Bellina

**Affiliations:** 1 Department of Pharmacy, University of Pisa, Via Bonanno, I-56126 Pisa, Italy; leonardo.carbonetti@farm.unipi.it (L.C.); simone.brogi@unipi.it (S.B.); vincenzo.calderone@unipi.it (V.C.); 2 Department of Chemistry and Industrial Chemistry, University of Pisa, Via Giuseppe Moruzzi 13, 56124 Pisa, Italyfabio.bellina@unipi.it (F.B.); 3 Interdepartmental Research Centre of Ageing Biology and Pathology, University of Pisa, Via Risorgimento 36, 56126 Pisa, Italy

**Keywords:** SIRT1, activators, cardioprotection, resveratrol, diarylimidazoles

## Abstract

Sirtuin 1 (SIRT1) is an NAD^+^-dependent deacetylase implicated in various physiological and pathological processes, including cardiovascular diseases. The lead compound for SIRT1, resveratrol (**1**), as well as natural-derived and synthetic SIRT1-activating compounds demonstrated to exert cardioprotective effects. In the present work, we evaluated a small series of diarylimidazoles, of which **4** emerged, in in vitro enzymatic assays, as an activator of SIRT1 endowed with a similar potency compared with that of **1**. Therefore, **4** was subjected to pharmacological investigation, where it was proven to reduce myocardial damage induced by ischemia/reperfusion injury in isolated rat hearts, thus demonstrating its cardioprotective properties. An in silico study suggested the binding mode of this derivative within SIRT1 in the presence of the p53-AMC-peptide. These promising results could pave the way to further expand and optimize this chemical class of new SIRT1 activators as potential cardioprotective agents.

## 1. Introduction

Sirtuins are a highly conserved family of nicotinamide adenine dinucleotide (NAD^+^)-dependent proteins implicated in the homeostatic regulation of numerous cellular activities, and in recent years, they have received great attention for their potential role under physiological and pathological conditions. Among the sirtuins, the most studied is undoubtedly SIRT1; indeed, considering its involvement in the pathophysiology of many human diseases, it has become a feasible target and many SIRT1-activating compounds have been currently identified and developed as potential therapeutic agents [1,2,3,4,5,6,7,8,9,10].

SIRT1 reversibly deacetylates the ε-acetyl-lysine residues of both histone and non-histone proteins by forming deacetylated targets in a two-step process. Specifically, the first step consists of NAD^+^ cleavage together with the covalent attachment of the ADP-ribose unit to the acetyl group of the protein target; in the second step, the hydrolysis of the acetyl-lysine bond generates the 2′-O-acetyl-ADP-ribose product [11]. The expression and/or activity of SIRT1 is progressively reduced during physiological ageing, leading to the onset of a variety of ageing-related diseases, including cardiovascular ones. In this regard, the role of SIRT1 in the protection against the damage induced by myocardial infarction has been widely demonstrated. Indeed, an overexpression of SIRT1 as well as the activation of SIRT1, by using pharmacological agents such as resveratrol and synthetic derivatives, can protect the myocardium against ischemia–reperfusion (I/R) events. Conversely, SIRT1 knock-out (SIRT1-KO) mice, exposed to I/R injury, showed a significantly increased damaged size. The SIRT1 stimulation is associated with an upregulation of antioxidant defences; an increase in FOXO1 and manganese superoxide dismutase (MnSOD) and a downregulation of pro-apoptotic pathways mediated by caspase 3 and Bax were reported [12].

Resveratrol (trans-3,5,4′-trihydroxystilbene, **1**), a polyphenol extracted from grape skins, red wine, and other edible materials, is probably the most relevant SIRT1-activating natural product. This stilbenoid derivative exerts cardioprotective activities by means of SIRT1 activation in various in vitro and in vivo models of myocardial I/R injury, and it has been demonstrated to increase lifespan in *Saccharomyces cerevisiae*, *Caenorhabditis elegans*, and *Drosophila melanogaster* [13].

Nevertheless, **1** is characterized by poor bioavailability due to extensive in vivo conversion into its sulphate and gluconate metabolites [14].

**Figure 1:**
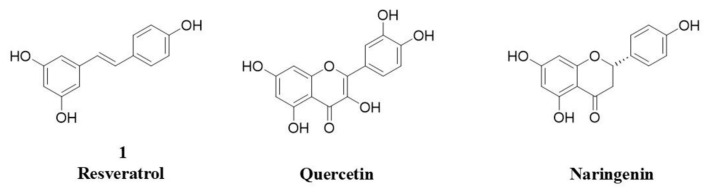
Chemical structures of natural-derived recognized SIRT1 activators.

Quercetin and naringenin are other examples of natural SIRT1 activators, and similarly to **1**, they are characterized by poor bioavailability (Figure 1) [15,16].

As regards synthetic SIRT1-activating compounds, numerous molecules have been developed by drawing inspiration from resveratrol (**1**), modifying its chemical–physical properties to improve its pharmacokinetic profile.

**Figure 2:**
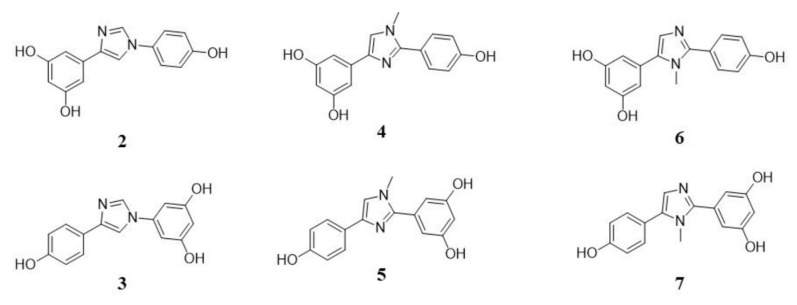
Chemical structures of the tested compounds.

With this aim in mind, in the present study we investigated a series of previously synthesized compound **1** analogues in which the stereochemically defined *trans* carbon–carbon double bond(s) have been conformationally locked within heteroaromatic rings, specifically based on an imidazole core (Figure 2) [17,18].

This structural modification was originally designed to improve the metabolic stability of the parent compound, addressing the chemical lability of **1** double bonds, which in vivo is prone to *trans*-to-*cis* isomerization and reduction to saturated derivatives, transformations that lead to biologically inactive metabolites [19,20]. Given the well-established role of **1** as a SIRT1 activator, these analogues, featuring a 1,3-relative arrangement of the aryl groups on the imidazole ring to mimic the *trans* geometry of the natural compound, have been subjected to biological evaluation, which revealed their capacity to modulate SIRT1 activity.

## 2. Results and Discussion

### 2.1. In Vitro Evaluation of Potential SIRT1 Activators

**Figure 3:**
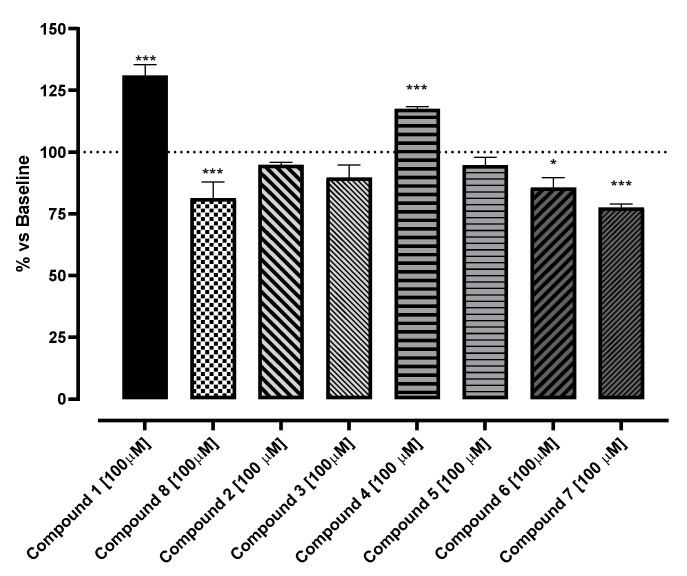
Stimulation of SIRT1 enzyme promoted by the selected compounds. SIRT1 activity is expressed as % vs. baseline (100%). The vertical bars symbolize the standard errors (*n* = 6). * indicates the significance of the compounds vs. the reference compound (**1**). * *p* < 0.05; *** *p* < 0.005.

The potential SIRT1 activators, resveratrol analogues, were tested using a SIRT1-specific enzymatic assay at a concentration of 100 µM, to compare their activity with reference compound **1** (Figure 1). According to the literature, **1** showed the profile of activator, increasing the activity of SIRT1 at 136 ± 0.1%; on the other hand, Sirtinol (**8**), the negative control, significantly reduced the SIRT1 activity at 81 ± 6.7% (Figure 3) [1,2,3,4,5]. Among the tested compounds, **4** was the only resveratrol analogue endowed with stimulating activity, increasing the activity of SIRT1 up 118 ± 1%. Conversely, **6** and **7** markedly reduced SIRT1 activity (86 ± 4 and 78 ± 1.5%, respectively). Compounds **2**, **3**, and **5** were unable to significantly influence the activity of the SIRT1 enzyme.

In summary, based on the in vitro test on isolated enzymes, this screening has identified three compounds able to significantly influence SIRT1 activity: two novel inhibitors candidates (**6** and **7**) and one compound (**4**) that behaves as an SIRT1 activator. Although this paper is focused on the identification of novel activators of SIRT1, possessing a better pharmacokinetic profile than **1**, in the future, **6** and **7** might be explored for their possible anti-proliferative activity. Regarding the compound endowed with stimulating properties, **4** was selected for further ex vivo and in silico steps of characterization.

### 2.2. Ex Vivo Evaluation of ***4***

**Figure 4:**
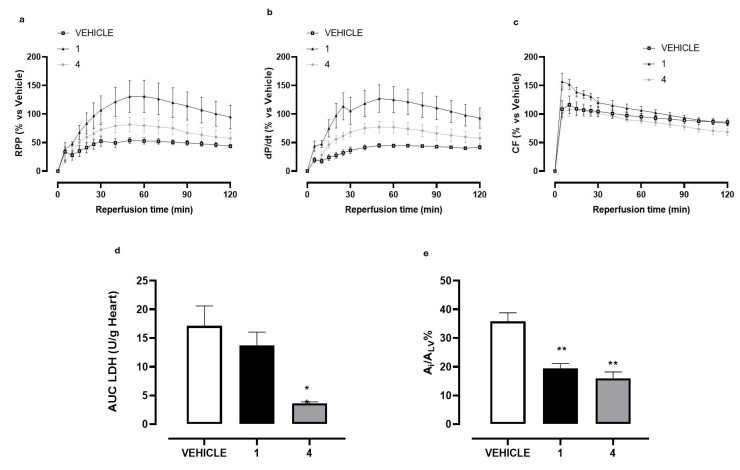
The graphs represent the functional, biochemical, and morphological changes induced by treatment of the hearts with **1** (resveratrol), **4,** or with their vehicle (DMSO 1%) before I/R episode. (**a**) Time-course of the changes in RPP% during the reperfusion; (**b**) time-course of the changes in dP/dt% during the reperfusion; (**c**) time-course of the changes in CF% during the reperfusion; (**d**) changes in LDH amount released in the reperfusion period (U/g heart); (**e**) changes in the percentage of ischemic area vs. left ventricle area (A_i_/A_LV_%). The vertical bars symbolize the standard errors (*n* = 6). Asterisks show a statistically significant difference from the value observed in the hearts of vehicle-treated animals (* *p* < 0.05; ** *p* < 0.01).

The functional efficacy of **4** was investigated in hearts isolated and perfused on Langendorff apparatus submitted to I/R protocol, which represents a well-known model to reproduce myocardial damage induced by ischemic events. In our experimental conditions, an I/R episode produced marked damage to the isolated hearts of vehicle-treated rats. In this regard, a marked decrease in the functional parameters of myocardial contractile function (RPP) was observed during the reperfusion time and at 60 min of reperfusion a compromised RPP value (52.85 ± 12.16%) was measured. The damage induced by the I/R event was also visible through the other functional markers (dP/dt and CF) and the morphometric analysis. In particular, the size of the ischemic area was equal to 35.76 ± 3.01%. Finally, the dosage of LDH levels in the perfusate confirmed the compromise of cardiac function. LDH was abundantly released in the perfusate during the first part of reperfusion, according to the literature [21,22]; the total amount at the end of reperfusion was 17.11 ± 3.48 U/g (Figure 4a–e).

As expected, 10 min of perfusion with **1** (10 µM) improved functional as well as morphometric and biochemical markers. At 60 min of the reperfusion period (corresponding at the peak of the maximal effect) the RPP value was significantly increased (130.80 ± 56.34%) and perfectly in line with the dP/dt value (124.91 ± 22.97%). Interestingly, **1** significantly increased the CF in the first part of the reperfusion period (for approximately 30 min). Although a mechanistic investigation was not carried out, compound **1**, apart from the stimulation on SIRT1 enzyme, acts on numerous intracellular targets [23,24,25,26]; in the specific of this experimental protocol, we can speculate that a such pharmacodynamic profile might be due to the well-known vasorelaxing effect of **1**, which might at least in part participate in cardioprotection. Consistently, a significantly reduced degree of damage (A_i_/A_LV_% = 19.40 ± 1.70%) and a trend of reduction in LDH released during the reperfusion period (13.74 ± 2.23 U/g) were also measured (Figure 4a–e).

Very interestingly, compound **4**, perfused at the same concentration of **1**, showed to significantly improve the functional parameters, both RPP and dP/dt, reaching after 60 min a value of 79.79 ± 20.45% and 77.00 ± 10.34%, respectively. Although these values are lower than those of **1**, the time-course curves during the reperfusion period are clearly indicative of a significant cardioprotective effect. Unlike **1**, no vasoactive effect was observed with the selected compound on the CF-time-course curve. Finally, **4** proved to significantly contain the A_i_/A_LV_ percentage (15.97 ± 2.23%), and to more evidently reduce LDH amount, when compared with **1**. Taken together, these results unequivocally demonstrate the cardioprotective activity of **4** against I/R injury.

### 2.3. Binding Mode of ***4*** Within SIRT1

**Figure 5:**
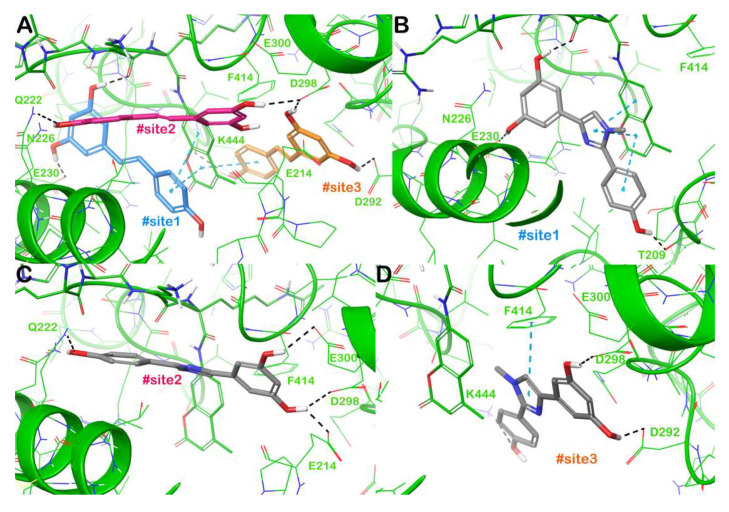
Binding mode of **1** within SIRT1 according to the crystal structure (PDB ID 5BTR), highlighting the interactions with the three different sites (panel (**A**)). Binding mode of **4** (grey sticks) within SIRT1 with particular focus on #site1, (panel (**B**)), #site2 (panel (**C**)), and #site3 (panel (**D**)). The p53-AMC-peptide is reported as small green thin sticks. Residues in the binding sites are represented by lines and hydrogen bonds are shown as grey dotted lines, whereas the π-π stacking is represented by cyan dotted lines. Images were generated using Maestro (Schrödinger, LLC, New York, NY, USA, 2020).

To investigate the potential interactions governing SIRT1 activation by **4**, we conducted a computational study using a previously described protocol [1,3,7]. Starting from the described mechanism of action of **1** in activating SIRT1, we compared the binding mode of **1** with that of **4**. In brief, **1** activates SIRT1 by simultaneously targeting three different binding sites located at the N-terminal domain (NTD) of the enzyme, two of which are necessary for SIRT1 activation, whereas the third site is defined as an accessory site with limited influence on activating SIRT1, as demonstrated by mutagenesis experiments [27]. As a result, the presence of two **1** molecules at the same moment facilitates an interaction with the p53-AMC-peptide and SIRT1 NTD and plays a crucial role in enhancing the binding affinity between the peptide and SIRT1, thereby increasing the enzyme’s activity. The binding mode of **1** within the three binding sites (defined as #site1, #site2, and #site3) is highlighted in Figure 5A. At #site1, **1** interacts with the sidechain of E230 via an H-bond, whereas further contacts (H-bonds and π-π stacking) were observed with the p53-AMC-peptide. Considering #site2, **1** can target Q222 and D298 via H-bonds. Finally, **1** in the accessory #site3 establishes H-bonds with D292, D298, and K444 and it can form hydrophobic contacts with the p53-AMC-peptide. Based on the molecular docking analysis, the proposed mode of interaction within SIRT1-binding sites accounted for a binding energy of −7.12 kcal/mol (#site1), −7.16 kcal/mol (#site2), and −7.21 kcal/mol (#site3). Regarding **4**, we observed a comparable interaction pattern when docked within SIRT1-binding sites with respect to those observed for **1**. Thus, **4** was able to target E230 (H-bond) and the p53-AMC-peptide (H-bonds and π-π stacking) at #site1, and in addition, we detected an H-bond with T209. This binding mode with an increased number of contacts, especially the strong network of π-π stacking with the p53-AMC-peptide, accounted for a more favourable docking score (−10.7 kcal/mol) with respect to the reference ligand **1** (Figure 5B). At #site2, **4** established a strong network of H-bonds targeting Q222, E214, D298, and E300, with a docking score of −8.79 kcal/mol, slightly higher than that of 1 (Figure 4c). Finally, at #site3, **4** was able to target the same residues of **1** (D292, D298, and K444 by H-bonds), with an additional π-π stacking with F414, and a binding energy of −7.84 kcal/mol (Figure 4d). The computational investigation revealed that the binding modes of **4**, as determined by molecular docking experiments, are consistent with SIRT1 activation, as demonstrated by in vitro experiments.

## 3. Materials and Methods

### 3.1. Diarylimidazole Synthesis

The synthesis of compound **1** analogues was performed as previously described [18] and reported in the Supporting Information. Briefly, 1,4-diaryl-*1H*-imidazoles **2** and **3** were obtained by demethylation of precursors prepared by a palladium-catalyzed Suzuki cross-coupling reaction involving commercially available arylboronic acids and 4-bromo-*1H*-imidazole, followed by a copper-catalyzed Buchwald N-arylation of the resulting 4-aryl-*1H*-imidazoles with the required aryl bromides. 2,4-Diaryl-1-methyl-*1H*-imidazoles **4** and **5** were obtained by demethylation of precursors achieved in the same conditions of the palladium-catalyzed Suzuki cross-coupling reaction involving commercially available arylboronic acids and 4-bromo-1-methyl-*1H*-imidazole, followed by a copper-catalyzed C2-arylation of the resulting 4-aryl-1-methyl-*1H*-imidazoles with the required aryl bromides. On the contrary, 2,5-diaryl-1-methyl-*1H*-imidazoles **6** and **7** were obtained by demethylation of the corresponding methyl ethers obtained by palladium and copper promoted sequential C5/C2 double arylations of 1-methyl-*1H*-imidazole with the required aryl bromides. All compounds, intermediates, and target molecules, were isolated by flash chromatography on silica gel. The spectroscopic data are in line with those reported previously [18]. GLC analyses showed that their chemical purity was higher than 97%.

### 3.2. In Vitro Assay

SIRT1 activity was analyzed by using a direct fluorescent screening assay kit (Item No. 10010401, Cayman Chemical, Ann Arbor, MI, USA), following the protocol user guide. Preliminarily, the interference of the compounds with the fluorophore and the developer were evaluated to select those compounds that gave interference values less than or equal to 10%. Compound **1** (Sigma-Aldrich, St. Louis, MO, USA) was used as a positive control and tested at the final concentration of 100 µM. All resveratrol analogues (**2**–**7**) were also tested at 100 µM to compare their activity on SIRT1 with **1**. The vehicle (Baseline) was a 1% DMSO–water solution. Finally, **8** (100 µM, Item No. 10523, Cayman Chemical, Ann Arbor, MI, USA) was used as SIRT1 inhibitor of reference and considered as a negative control. Fluorescence was analyzed with the EnSpire spectrofluorometer (PerkinElmer, Waltham, MA, USA) at an excitation wavelength of 350–360 nm and an emission wavelength of 450–465 nm. An increase in recorded fluorescence was directly proportional to the activation of SIRT1, while a decrease in the fluorescence was indicative of an inhibition of SIRT1. Data obtained were analyzed by removing background and normalizing the fluorescence value of the vehicle, considered as 100% of the enzyme activity. The effects of the potential SIRT1 activators were statistically analyzed by one-way ANOVA followed by a Bonferroni post-test. A *p*-value < 0.05 was considered indicative of a significant difference.

### 3.3. Ex Vivo Studies

The experiments were carried out in accordance with the European Union Council Directive 2010/63/EU on male normotensive Wistar Kyoto rats (350–400 g). The experimental protocol was previously authorized by the Ethical Committee of the University of Pisa and of the Italian Ministry of Health (authorization number DB173.N.IXS). All the animals were housed in a room under a controlled temperature (23–25 °C) and humidity (50%) and they were exposed to 12 h:12 h light/dark cycles, with food and water provided ad libitum. On the day of the experiment, a solution of sodium thiopental was injected in the rat at a dosage of 100 mg/kg (i.p.) and, once the animal was anesthetized, the axillary artery was severed to remove blood. After opening the rib cage, hearts were quickly excised and placed in a 4 °C Krebs solution (composition mM: NaHCO_3_ 25.0, NaCl 118.1, KCl 4.8, MgSO_4_ 1.2, CaCl_2_·2H_2_O 1.6, KH_2_PO_4_ 1.2, and glucose 11.5) equilibrated with clioxicarb (a gas mixture composed by 95% plus O_2_ 5% CO_2_), to stop the contraction and reduce oxygen consumption. The ascending aorta was cannulated; each heart was placed in a Langendorff apparatus and perfused under constant pressure (70–80 mmHg) with Krebs solution, maintained at 37 °C and bubbled continuously with clioxicarb. The above procedure was completed within 2 min. A water-filled latex balloon connected to a pressure transducer (Bentley Trantec, mod 800) was introduced into the left ventricle through the mitral valve and the volume was adjusted to achieve a stable left ventricular end-diastolic pressure of 5–10 mmHg; this balloon allowed us to record the heart’s functional parameters. Heart rate (HR), left ventricular developed pressure (LVDP), and dP/dt (i.e., the ratio of pressure changes in the ventricular cavity during the isovolemic contraction period) were continuously monitored by Biopac software 2.0 (Goleta, CA, USA); the rate pressure product (RPP) was calculated as RPP = LVDP × HR. After 20 min of equilibration, the hearts were perfused for 10 min with a 0.1% DMSO–water solution (vehicle), with a 10 µM solution of **1** as a positive control [28,29,30] or with **4** (10 µM), the selected compound after the in vitro screening. A period of 30 min of global ischemia followed the equilibration phase. At the end of the ischemic period, the hearts were re-perfused for 120 min with Krebs solution. During the equilibration and the re-perfusion periods, the coronary flow (CF) was measured volumetrically by collecting the perfusate every 5 min until the 60th min of re-perfusion and every 10 min until the end of the experiment. The perfusate was also collected to evaluate the activity of the LDH enzyme, a known biochemical marker of ischemic damage, and was spectrophotometrically measured. LDH was assessed by adding 27.6 mM pyruvate and 4.8 mM NADH and measuring the conversion of NADH to NAD+ at λ = 340 nm in kinetics for 10 min. The amount of released LDH has been expressed in U/g in 120 min of reperfusion, resulting from the AUC analysis (area under the curve of the LDH amount recorded) and related to 1 g of the heart weight. During the reperfusion period, functional parameters (CF, RPP and dP/dt) were measured and expressed as a percentage of the pre-ischemic period. At the end of the 120 min of re-perfusion, the heart was removed from the Langendorff apparatus, dried, weighed, and subsequently the left ventricle was isolated. This was cut in slices of about 2 mm and immersed in a 1% *w*/*v* solution of 2,3,5 triphenyltetrazolium chloride (TTC, Sigma-Aldrich) and dissolved in a phosphate buffer (pH = 7.4) for 20 min in the dark and at 37 °C. TTC reacts with dehydrogenases present in intact and viable cells and oxidizes to formazan, a red and insoluble compound. Then, slices were fixed in a 10% *v*/*v* formaldehyde–water solution. Subsequently, the ventricular slices were photographed and analyzed to identify the necrotic areas (visible as a white or light pink colour) and the healthy areas (visible as a strong red due to the TTC reaction). It was possible to calculate the ischemic area as a percentage of the total area of the left ventricle through photographs of the sections subjected to planimetric analysis carried out 24 h after the reaction with the TTC (A_i_/A_LV_).

All values are expressed as a mean ± standard error for six different experiments. The effects of **1** and **4** on Ai/ALV and LDH were statistically analyzed by Student’s *t*-test, while their effects on RPP, dP/dt, and CF were evaluated by two-ways ANOVA test. *p* < 0.05 was considered indicative of a significant difference.

### 3.4. Computational Details

Diarylimidazoles **2**–**7** were built and prepared as described using the tools available in Maestro [31,32,33,34]. The compounds were minimized utilizing MacroModel (MacroModel release 2020, Schrödinger, LLC, New York, NY, USA, 2020) and prepared using LigPrep (LigPrep release 2020, Schrödinger, LLC, New York, NY, USA, 2020) to establish the most suitable ionization state at physiological pH). The 3D structure of SIRT1 was downloaded from PDB (PDB ID 5BTR) [23] and prepared using the protein preparation wizard tool available in the Schrödinger suite, as reported [1,3,34]. Molecular docking experiments were performed using Glide software (Glide release 2020, Schrödinger, LLC, New York, NY, USA, 2020) with the SP-scoring function, as previously described [1,3]. The activation mechanism of SIRT1, as explained for **1** and visualized by the crystal structure in which **1** is bound to SIRT1 at three binding sites, prompted us to create energy grids for docking. These grids were prepared using a standard protein atom-scaling factor of 1.0 Å, with a cubic box centred on the crystallized molecule. We then prepared a custom grid for each selected binding site. Following grid generation, the compounds were docked into SIRT1, taking into account its three distinct binding sites. The post-docking minimization step considered 1000 docked poses and used the Glide SP docking score (GlideScore) for evaluation. The SP-scoring function showed a capability to correctly accommodate **1** into its three binding sites with a low RMSD [1].

## 4. Conclusions

SIRT1 enzyme has been investigated because of its potential to develop new therapeutic tools for the prevention/treatment of several age-related chronic diseases, including myocardial infarction. Although **1** is considered the best lead compound in in vitro assays, in the in vivo studies it has failed because of the poor bioavailability of this polyphenol. Therefore, the synthesis of new molecules with improved pharmacokinetic profiles with respect to **1** could fill this gap. In this context, a small series of **1** analogues was evaluated on an isolated SIRT1 enzyme to enhance SIRT1 activation.

Among these molecules, **4** showed an SIRT1 activation capacity similar to that of resveratrol and had a similar ex vivo cardioprotective profile, demonstrating the capability of **4** to improve functional and morphometric parameters, as well as its function as a biochemical marker of myocardial injury. Finally, the in silico approach revealed that the binding modes of **4** are consistent with SIRT1 activation, as observed by in vitro experiments.

Further studies will undoubtedly be necessary to implement the knowledge on the pharmacodynamic and pharmacokinetic profiles of the compound. However, these preliminary results suggest that **4** can be considered a valid candidate as an SIRT1 activator, and it may be worth investing in its characterization and the design of its derivatives, paving the way for a new class of SIRT1 activators potentially interesting for the treatment/prevention of cardiovascular diseases. Indeed, besides the cardioprotection against I/R damage, the SIRT1 enzyme plays crucial roles in other cardiovascular and cardiometabolic diseases and more generally in several ageing-related disorders. In the future, these fields will be evaluated with selected molecules.

## Data Availability

Dataset is available on request from the authors.

## Supplementary Materials

The following supporting information can be downloaded at: https://www.mdpi.com/article/10.3390/molecules30224378/s1, Synthetic Protocol of compounds **2**–**7**.
