# Supplementary material for: Pharmacological Investigation of a Novel Resveratrol-like SIRT1 Activator Endowed with a Cardioprotective Profile
Source: Molecules. 2025 Nov 13;30(22):4378. doi: 10.3390/molecules30224378 (PMC12655221; doi:10.3390/molecules30224378)
Supplement: Supplementary file 1 [file molecules-30-04378-s001.zip › molecules-3899555-supplementary.pdf]

Synthetic Protocol of compounds **2-7**

General

Melting points were recorded on a hot-stage microscope (Reichert Thermovar). Aldrich precoated 60 F254 aluminum silica gel sheets were used for TLC analyses. GLC analyses were performed using two types of capillary columns: an Alltech AT-35 bonded FSOT column (30 m 0.25 mm i.d.) and an Alltech AT-1 bonded FSOT column (30 m 0.25 mm i.d.). Purifications by flash chromatography were performed using silica gel Aldrich 60 (particle size 0.040e0.063 mm). EI-MS spectra were measured at 70 eV by GLC/ MS. NMR spectra were recorded at room temperature at 200 MHz (1H) and 50.3 MHz (13C) and were referred to TMS or to the residual protons of deuterated solvents. 4*(5)*-Bromo-*1H*-imidazole , 4-methoxyphenylboronic acid , 3,5-dimethoxyphenylboronic acid, 1-bromo-4-methoxybenzene, 1-bromo-3,5-dimethoxybenzene, 4-bromo-1-methyl-*1H*-imidazole, benzyltriethylammonium chloride, tetrabutylammonium acetate, copper(I) trifluomethanesulfonate toluene complex, 1,10-phenanthroline, $PdCl_2$(dppf), palladium acetate, copper(I) iodide, and cesium fluoride were commercially available and, unless otherwise stated, were used as received. 1-methyl-*1H*-imidazole was purified by vacuum distillation. Unless otherwise stated, commercially anhydrous solvents were used as received. Dichloromethane was anhydrified by distillation over CaH2.

Synthetic general procedures

The synthesis of 1,4-,2,4-, and 2,5-diarylimidazoles, in which 3,5-dihydroxyphenyl and 4-hydroxy phenyl are substituted alternately in the relative positions, have been synthetized according to our previous procedure and is reported in scheme 1 (https://doi.org/10.1016/j.tet.2015.02.024).

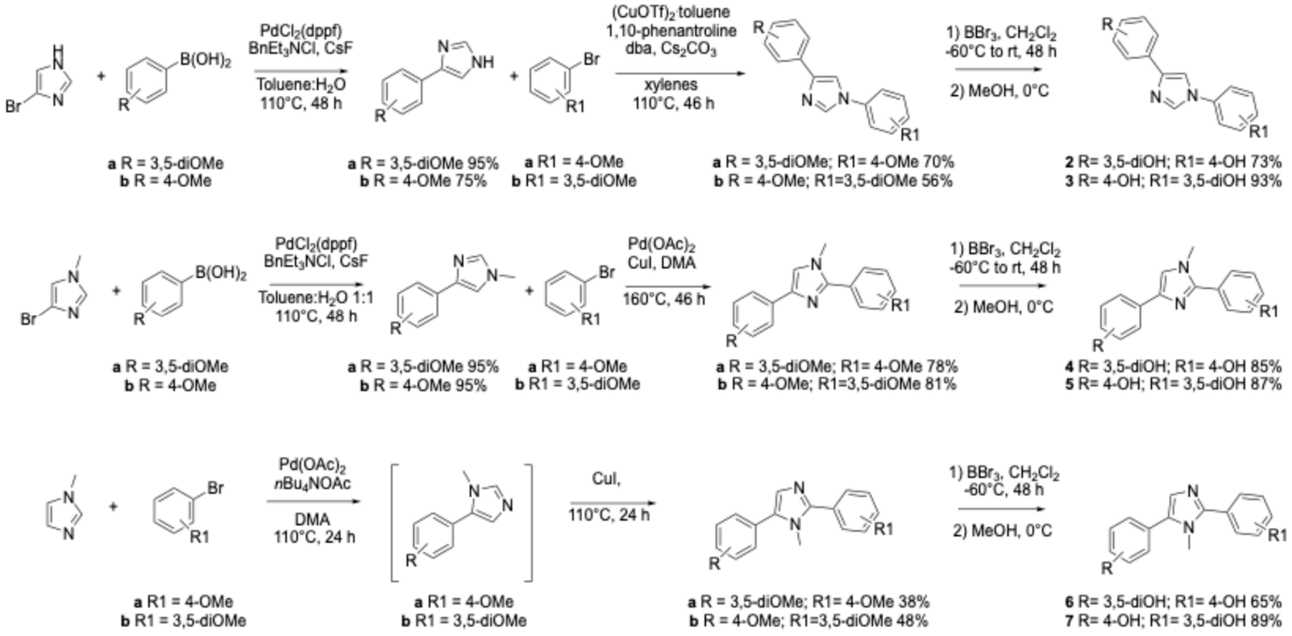

Scheme 1. Synthetic protocols to obtain compounds **2-7.**

1. **General procedure of Suzuki–Miyaura cross-coupling to obtain 4(5)-aryl-*1H*-imidazole and 4-aryl-1-methyl-*1H*-imidazoles**

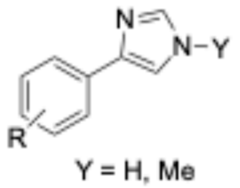

A deaerated mixture of 4*(5)*-bromo-*1H*-imidazole (1 equiv) or 4-bromo-1-methyl-*1H*-imidazole (1 equiv), arylboronicacid (2 equiv), CsF(4 equiv), $PdCl_2$(dppf) (5% mol), and $BnEt_3NCl$ (5% mol) in toluene and water 1:1 was heated at reflux under argon for 48 h. The mixture was then cooled to room temperature and partitioned between water and AcOEt, and the organic extract was dried and concentrated under reduced pressure. The residue was purified by flash chromatography on silica gel to provide the desired products with a yield up to 90%. Product spectroscopic data are in line with those reported in our previous work (https://doi.org/10.1016/j.tet.2015.02.024). These products were then arylated in position 1 or 2 according to the N-H or C-H arylation procedure to obtain the methylated precursors of compounds **2-5**.

2. **General procedure for the synthesis of 1,4-diaryl-*1H*-imidazoles by Buchwald N-arylation**

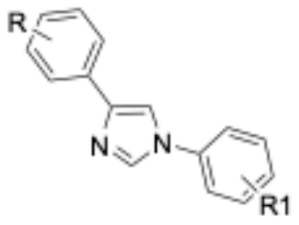

To a flame-dried reaction vessel were added 4*(5)*-arylimidazole (1 equiv), 1,10-phenanthroline (1 equiv), *trans, trans*-dibenzylidene acetone (dba) (5% mol), Cs_2CO_3 (1.1 equiv), and copper(I) trifluomethanesulfonate toluene complex (5% mol). The reaction vessel was fitted with a silicon septum, evacuated, and back-filled with argon, and this sequence was repeated twice. Xylenes (1mL) and aryl bromide (1.5 equiv) were then added successively under a stream of argon by syringe at room temperature. The resulting mixture was stirred under argon at 110°C until GLC analysis showed that the reaction was complete (48h). The resultant heterogeneous mixture was allowed to cool to room temperature, diluted with EtOAc, filtered through a plug of silica gel, and eluted with additional EtOAc. The filtrate was concentrated under reduced pressure, and the residue was purified by flash chromatography on silica gel to provide the products: methylated analogues of **2** and **3** with yields up to 60%. Product spectroscopic data are in line with those reported in our previous work (https://doi.org/10.1016/j.tet.2015.02.024).

3. **General procedure for the synthesis of 2,4-diaryl-1-methyl-*1H*-imidazoles by direct C2 arylation**

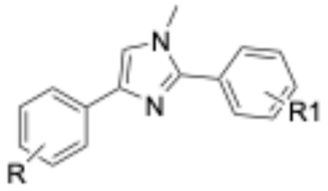

4-aryl-1-methyl-*1H*-imidazoles (1 equiv), $Pd(OAc)_2$ (5% mol), CuI (2 equiv) were placed in the reaction vessel under a stream of argon. The reaction vessel was fitted with a silicon septum, evacuated, and back-filled with argon, and this sequence was repeated twice. Aryl bromide (1.50 equiv) and deaerated DMA (5mL) were then added by syringe under a stream of argon at room temperature, and the resulting mixture was stirred at 160°C under argon. The degree of completion of the reaction was established by GLC and GLC/MS analysis of a sample of the crude reaction mixture after treatment with a saturated aqueous NH_4Cl solution and

extraction with AcOEt. After being cooled to 20°C, the reaction mixture was diluted with AcOEt and poured into a saturated aqueous NH_4Cl solution. The resulting mixture was basified with a few drops of aqueous NH_4OH, stirred in the openair for 1 h, and then extracted with AcOEt. The organic extract was washed with water, dried, and concentrated under reduced pressure, and the residue was purified by flash chromatography on silica gel. This procedure was employed to prepare 2,4-diaryl-1-methyl-*1H*-imidazoles, methylated precursors of **4** and **5** in yields close to 80%. Product spectroscopic data are in line with those reported in our previous work (https://doi.org/10.1016/j.tet.2015.02.024).

4. General procedure for the synthesis of 2,4-diaryl-1methyl-*1H*-imidazoles by sequential one-pot C5 and C2 direct arylations

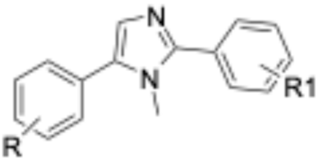

$Pd(OAc)_2$ (5% mol) and Bu_4NOAc (2 equiv) were added to a flame-dried reaction vessel. The reaction vessel was fitted with a silicon septum, evacuated, and back-filled with argon. This sequence was repeated twice. DMA (5 mL), aryl bromide (1 equiv), and 1-methyl-*1H*-imidazole (1.1 equiv) were then added successively under a stream of argon by syringe at room temperature. The resulting mixture was stirred at 110°C under argon for 24 h. CuI (2 equiv) and the other aryl bromide (1.5 equiv) were then sequentially added to the resulting brown solution under a stream of argon. The reaction mixture was heated to 110°C and stirred at this temperature for 24h. After cooling to room temperature, the reaction mixture was diluted with AcOEt and poured into a saturated aqueous NH_4Cl solution. The resulting mixture was basified with a few drops of aqueous NH_4OH, stirred in the open air for 1h, and then extracted with AcOEt. The organic extract was washed with water, dried, and concentrated under reduced pressure, and the residue was purified by flash chromatography on silica gel. This procedure was employed to prepare 2,5-diaryl-1-methyl-*1H* imidazoles, methylated analogues of **6** and **7** with yields close to 40%. Product spectroscopic data are in line with those reported in our previous work (https://doi.org/10.1016/j.tet.2015.02.024).

5. General procedure for the synthesis of polyphenolic imidazole-based analogues of 1 by demethylation with BBr3

To a solution of O-methoxyphenyl imidazoles (1 equiv) in dry CH_2Cl_2 (10 mL/mmol), which was stirred at -60°C, was added a 1 M solution of BBr_3 in CH_2Cl_2 (9 equiv). The reaction mixture was allowed to warm up to room temperature and stirred at this temperature for 48 h. After cooling to 0°C, the reaction mixture was diluted with methanol (1.5 mL), and AcOEt (10 mL) and a 10% aqueous solution of NaOH (10 mL) were sequentially added. The organic phase was recovered and acidified with a 10% solution of HCl cooled at 0°C. The formed precipitate was collected by filtration and dried in vacuo, to achieve chemically pure title compounds. This procedure affords the desired products of Figure 1 in yields up to 60%.

Product spectroscopic data are in line with those reported in our previous work (https://doi.org/10.1016/j.tet.2015.02.024).
